# Characterization of Plan Complexity and Its Role in Quality Assurance for AI-Assisted CBCT-Based Online Adaptive Radiotherapy of Prostate Cancer

**DOI:** 10.3390/cancers18101557

**Published:** 2026-05-11

**Authors:** Antonio Giuseppe Amico, Sonia Sapignoli, Samuele Cavinato, Badr El Khouzai, Marco Andrea Rossato, Marta Paiusco, Chiara Paronetto, Alessandro Scaggion, Matteo Sepulcri, Andrea Bettinelli

**Affiliations:** 1 Medical Physics Department, Veneto Institute of Oncology IOV-IRCCS, 35128 Padova, Italy; antonio.amico@iov.veneto.it (A.G.A.); sonia.sapignoli@iov.veneto.it (S.S.); marcoandrea.rossato@iov.veneto.it (M.A.R.); marta.paiusco@iov.veneto.it (M.P.); andrea.bettinelli@iov.veneto.it (A.B.); 2 Radiation Oncology Department, Veneto Institute of Oncology IOV-IRCCS, 35128 Padova, Italy; badr.elkhouzai@iov.veneto.it (B.E.K.); chiara.paronetto@iov.veneto.it (C.P.); matteo.sepulcri@iov.veneto.it (M.S.)

**Keywords:** online adaptive radiotherapy, plan complexity, statistical process control, patient-specific quality assurance, prostate cancer

## Abstract

Background/Objectives: Online adaptive radiotherapy (oART) generates plans at each fraction by exploiting AI-assisted optimization engines without explicit user control over modulation. This process challenges quality assurance since measurement-based Patient Specific Quality Assurance (PSQA) cannot be performed daily. This study aimed: (i) to characterize plan complexity in IOE-generated plans for prostate cancer using a reproducible set of PCMs, including the decomposition of inter-patient and intra-patient variability sources; (ii) to evaluate the association between PCMs and delivery accuracy within a cohort-informed SPC framework validated through leave-one-patient-out cross-validation; (iii) to investigate whether inter-fraction anatomical variations explain the observed plan complexity patterns, or whether complexity is predominantly an intrinsic signature of the AI-assisted optimizer. Methods: Twenty-one prostate cancer patients treated on a CBCT-based oART platform were retrospectively analyzed across three anatomical targets: prostatic bed (PrB), prostate (Pr), and prostate with seminal vesicles (PrSV). Six PCMs, namely MU/cGy, Modulation Complexity Score (MCS), Aperture Area Variability (AAV), Leaf Sequence Variability (LSV), Average Leaf Gap (ALG) and Plan Irregularity, were extracted. Additionally, five anatomical metrics (AMs) were computed from daily contours. Linear mixed-effects models (LMEMs) compared reference/online plans, decomposed variance via intraclass correlation coefficients (ICCs), and assessed PCM–gamma passing rate (GPR) associations. Leave-one-patient-out cross-validation (LOPO-CV) evaluated SPC threshold stability. The relationships between PCMs and AMs were investigated using LMEMs. Results: The AI-assisted optimization engine generated plans characterized by elevated monitor unit demand (average MU/cGy ≥ 6.8 ± 0.9) and narrow MLC apertures (ALG ≤ 17.7 mm ± 1.9 mm). No complexity differences emerged between offline and online-adapted plans, nor between anatomical targets. All PCMs showed significant associations with global GPR (*p* ≤ 0.027), though marginal R^2^ remained low (≤0.122). Notably, GPR dispersion increased systematically at higher complexity values, indicating that highly modulated plans exhibit reduced delivery predictability. LOPO-CV demonstrated stable tolerance/action limits. Anatomical variations explained less than 35% of the total variance in PCMs. Conclusions: Plan complexity in oART reflects the optimization paradigm and patient-specific anatomy rather than daily adaptation. PCMs can serve as surveillance indicators flagging high-risk fractions to support SPC-based monitoring.

## Simple Summary

Modern online adaptive radiotherapy (oART) systems exploit artificial intelligence (AI)-based workflows to automatically adjust treatment plans to a patient’s daily anatomy. However, during the whole process, the patient lies on the couch, and this creates a quality assurance challenge, since each session generates a new plan that cannot be verified through traditional physical measurements before delivery. In this study, we analyzed 451 treatment plans from 21 prostate cancer patients to understand what drives plan complexity and whether mathematical indicators can predict delivery accuracy. Our results demonstrate that the high complexity of these plans is an intrinsic characteristic of the AI-based algorithm, rather than a response to the patient’s daily anatomical variations. Although complexity metrics alone cannot replace direct checks or perfectly predict the accuracy of a single plan, highly complex treatments exhibit greater unpredictability and instability. Therefore, these indicators can be used to flag patients or treatment sessions at a higher risk of delivery inaccuracies in real-time, helping clinics prioritize their QA efforts based on plan-level risk indicators.

## 1. Introduction

Online adaptive radiotherapy (oART) enables daily replanning to match the anatomy of the day, which strongly relies on an AI-assisted workflow. However, the online workflow introduces quality assurance challenges that differ fundamentally from those of conventional radiotherapy. On the Varian Ethos platform, a new treatment plan is automatically generated at each fraction by the Intelligent Optimization Engine (IOE), an AI-assisted, directive-based, derivative-free optimizer in which the operator defines clinical objectives and priorities while the algorithm autonomously determines the modulation degree and MLC sequencing parameters [1,2,3,4]. Unlike conventional planning, where the physicist usually retains explicit control over optimization constraints and can iteratively adjust the balance between plan complexity and dosimetric quality, the IOE operates as a closed system: the internal logic governing how competing clinical goals are translated into fluence modulation is not transparent to the user, and the resulting plan complexity cannot be directly constrained. This opacity raises a specific concern, namely, whether the automatically generated plans maintain consistent deliverability across fractions. This cannot be addressed through conventional measurement-based patient-specific quality assurance (PSQA), since each adapted plan is generated for immediate delivery while the patient remains on the treatment couch [2,5,6,7].

Plan complexity metrics (PCMs) are mathematical descriptors that quantify aspects of MLC modulation, aperture geometry, and delivery demand; their role in characterizing plan quality has been increasingly recognized, with complexity proposed as a fundamental component of plan quality alongside dosimetric accuracy and robustness [8] and dedicated systematic reviews collecting the available indices [9,10]. Brooks et al. demonstrated how specific PCMs predict the impact of beam modeling errors on dose accuracy [11], and Cavinato et al. [12] explicitly showed the role of PCMs in identifying the optimal balance between dosimetric accuracy and clinical acceptability. From a QA perspective, O’Daniel et al. framed the failure modes that PSQA should detect [13], and Russo et al. demonstrated that statistical process control (SPC) Tolerance Limits (TLs) and Action Limits (ALs) should account for complexity-dependent variability in gamma passing rates, as recommended by AAPM TG-218 [5,14]. However, these tools have been developed and validated almost exclusively for workflows where the operator controls the optimization process—a condition that does not hold for AI-assisted engines such as the IOE.

To date, their application to oART remains limited. Zhao et al. suggested that measurement-based verification may not be needed at every Ethos fraction [15], and similar observations have been reported for MR-guided platforms [12,16,17,18]. However, no published study has specifically characterized how the IOE’s autonomous optimization behavior handles plan complexity across the adaptive process. It remains unclear whether the modulation patterns produced by the IOE reflect responses to daily anatomical variation or intrinsic properties of the AI-assisted optimizer itself, and whether PCMs can serve as a basis for delivery quality monitoring when per-fraction measurement-based PSQA is not feasible.

The aims of this study are the following: (i) to characterize plan complexity in IOE-generated plans for prostate cancer using a reproducible set of PCMs, including the decomposition of inter-patient and intra-patient variability sources; (ii) to evaluate the association between PCMs and delivery accuracy within a cohort-informed SPC framework validated through leave-one-patient-out cross-validation; (iii) to investigate whether inter-fraction anatomical variations explain the observed plan complexity patterns, or whether complexity is predominantly an intrinsic signature of the AI-assisted optimizer.

## 2. Materials and Methods

### 2.1. Ethos System and oART Workflow

The Varian Ethos version 1.0 is a software platform that enables oART on the Halcyon delivery system (Varian Medical Systems, Palo Alto, CA, USA), an O-ring linac delivering single-energy 6 MV flattening-filter-free (6X-FFF) photons with a maximum dose rate of 800 MU/min and jawless beam shaping obtained through a dual-layer MLC. Dose calculation is performed using the Acuros XB (AXB) algorithm with heterogeneity correction and dose-to-medium reporting [1].

The Ethos oART workflow follows the previously reported pipeline [1]: CBCT acquisition, AI-assisted contouring of influencer structures (reviewed and edited by the operator), synthetic CT generation, IOE-driven re-optimization according to the predefined directive hierarchy and final selection between the adapted and scheduled plan for delivery. Notably, the IOE determines modulation degree and MLC sequencing internally; these parameters cannot be directly constrained by the operator [2,4,19].

### 2.2. Study Cohort and Treatment Delivery

This retrospective study included 21 prostate cancer patients enrolled in the oART program treated between January 2022 and December 2023. Patients were classified into three anatomical target groups, hereafter abbreviated as: prostatic bed (PrB), prostate without seminal vesicles (Pr) and prostate with seminal vesicles (PrSV). The fractionation schemes and plans are summarized in Table 1. All plans were delivered using a 9-field sliding-window IMRT arrangement with equally spaced gantry angles (40° spacing). A collimator angle of 10° was used for all fields in all plans. This geometry was selected over VMAT to prioritize a more time-efficient online re-optimization process. This choice was crucial to maintain the total calculation and re-optimization time within approximately 5 min, thereby limiting the overall adaptive workflow duration to 15–20 min and avoiding bladder filling changes that could compromise dosimetric accuracy.

### 2.3. Plan Complexity and Anatomical Metrics

#### Plan Complexity Metrics

To characterize the complexity of the IOE-generated plans, six IMRT-relevant PCMs were extracted from each plan using the publicly available UCoMX (Universal Complexity Metrics Extractor) software package [20]. The PCMs were selected a priori from over forty descriptors implemented in UCoMX, with the objective of obtaining a metric set that captures complementary aspects of plan modulation, aperture geometry, and MLC sequencing. The rationale for this selection approach follows the methodology described by Cavinato et al. (2025), who demonstrated the importance of tailoring the metric panel to the delivery technique in order to avoid redundant information and improve interpretability [21]. The six selected metrics are the following:

- MU/cGy (normalized monitor units): Overall modulation and delivery demand;
- LSV (Leaf Sequence Variability): Difference in position between adjacent leaves;
- AAV (Aperture Area Variability): Variability in aperture size across control points;
- MCS (Modulation Complexity Score): Product of the LSV and AAV;
- ALG (Average Leaf Gap): Average separation between opposing leaves;
- PI (Plan Irregularity): Aperture shape irregularity.

Details of PCM calculation are available at https://zenodo.org/records/15673571 (accessed on 3 May 2026).

### 2.4. Patient-Specific Quality Assurance

In routine clinical practice, measurement-based PSQA is performed for the reference plan using an ArcCHECK diode array (Sun Nuclear Corp., Melbourne, FL, USA). For subsequent adapted fractions, delivery safety is monitored via independent secondary dose calculation (Mobius3D, Varian Medical Systems).

To assess whether the dosimetric performance of the reference plan is representative of the adapted plans that follow, a supplementary measurement-based PSQA was performed on a subset of five adapted fractions per patient (see Supplementary Materials for more details).

The γ-index passing rates (GPRs) were calculated using a 3%/2 mm criteria, 10% dose threshold, using both global—GPR (3%Global, 2 mm)—and local—GPR (3%Local, 2 mm)—normalization, in accordance with TG-218.

### 2.5. Anatomical Metrics

To evaluate the association between inter-fraction anatomical variations and plan complexity, five anatomical metrics (AMs) were extracted, namely PTV and CTV volumes in cm^3^, PTV–rectum and PTV–bladder intersection volumes in cm^3^ (called Rectum ∩ PTV and Bladder ∩ PTV, respectively), and the percentage overlap between the PTV and all surrounding OARs (Total Overlap, in percentage). All volumes were computed from the DICOM RT Structure files associated with the sCT using the voxel-counting method implemented in S-IBEX [22], a radiomic feature-extraction tool compliant with the Imaging Biomarker Standardization Initiative (IBSI) [23].

### 2.6. Statistical Analysis

The study design involved multiple adapted plans generated for the same patient, one at each treatment fraction. This setting differs from conventional IGRT protocols, where the same plan is delivered throughout the whole treatment course. This structure introduces statistical dependencies, with plans from the same patient sharing both anatomical and plan-related characteristics. To properly account for these dependencies and to separately quantify relationships at the population level (between patients) and at the individual level (within patients/across fractions), linear mixed-effects models (LMEMs) were applied.

All statistical analyses were performed in Python v3.12.12 using NumPy, Pandas, SciPy, Statsmodels, and Pingouin. Statistical significance was set at = 0.05 (two-sided, with Benjamini–Hochberg false discovery rate, BH-FDR, correction applied where appropriate).

#### 2.6.1. Assessment of Plan Complexity Across the Adaptive Process

Statistical differences in the dataset were assessed, accounting for both the planning phase (Reference vs. Online) and the anatomical target (i.e., PrB, Pr, or PrSV). In particular, an LMEM with the following mathematical formulation was adopted:

(1) $${PCM}_{ij}=\beta_{0}+\beta_{1}{\left(\mathrm{Planning}\;\mathrm{Phase}\right)}_{ij}+\beta_{2}{\left(\mathrm{Anatomical}\;\mathrm{Target}\right)}_{j}+u_{0j}+\varepsilon_{ij}$$

where $PCM_{ij}$ is the metric value for fraction i of patient j. Planning phase and anatomical target were included as fixed effects, with “Reference” and “PrB” set as their respective reference categories. The patient-specific random intercept $u_{0j}∼N\left(0,\sigma_{u}^{2}\right)$ accounts for the statistical dependence between fractions of the same patient; $\varepsilon_{ij}∼N\left(0,\sigma_{\varepsilon }^{2}\right)$ represents the residual error. The Intraclass Correlation Coefficient (ICC), defined as the ratio of between-patient variance to total variance (the sum of between- and within-patient variances), was calculated to determine whether the observed variability was predominantly patient-driven or fraction-driven.

#### 2.6.2. PSQA Assessment and SPC Framework

Process-based TLs and ALs on the GPRs were calculated using the SPC framework proposed in the AAPM TG-218 report. In particular, Equations (3) and (6) of the report were applied, setting T=100% and β=6. The process-based limits were derived separately for global and local GPRs.

Due to the absence of an historical IMRT database for this specific platform, the stability of the PSQA process was evaluated by applying the described approach to the plans in the dataset of this work. The generalizability of the derived TLs and ALs was assessed through leave-one-patient-out cross-validation (LOPO-CV), where one patient was held out, and limits were re-estimated on the remaining cohort at each iteration. This design emulates the prospective use case of applying control limits to an unseen patient [24].

#### 2.6.3. Relation Between Plan Complexity and Delivery Accuracy

We investigated the relationship between PCMs and GPR using the following univariate LMEM:

(2) $${GPR}_{ij}=\beta_{0}+\beta_{1}{\mathrm{PCM}}_{ij}+u_{0j}+\varepsilon_{ij}$$

where $PCM_{ij}$ and $GPR_{ij}$ are the PCM and GPR of interest for fraction i of patient j. Model fit was summarized using marginal R^2^ ($R^{2}m$) and conditional R^2^ ($R^{2}c$) following Nakagawa and Schielzeth (2013) [25]. $R^{2}m$ quantifies the proportion of total variance explained by the fixed effects alone (i.e., the PCM in this model), representing the model’s generalizable predictive capacity across unseen patients. $R^{2}c$ additionally incorporates the variance explained by the patient-specific random intercept ($u_{0j}$); the difference $R^{2}c$ − $R^{2}m$ therefore reflects the contribution of patient-level factors not captured by the predictor.

#### 2.6.4. Assessment of Anatomical Variations Across the Adaptive Process

Statistical differences in anatomical metrics (AMs) were assessed using the same LMEM formulation described in Section 2.6.1. The model accounted for both the Planning Phase and Anatomical Target as fixed effects, with a patient-specific random intercept ($u_{0j}$) to handle the nested structure of the data:

(3) $${AM}_{ij}=\beta_{0}+\beta_{1}{\left(\mathrm{Planning}\;\mathrm{Phase}\right)}_{ij}+\beta_{2}{\left(\mathrm{Anatomical}\;\mathrm{Target}\right)}_{j}+u_{0j}+\varepsilon_{ij}$$

#### 2.6.5. Relation Between Plan Complexity and Anatomy

The relationship between PCMs and AMs was assessed using the following univariate LMEM:

(4) $${PCM}_{ij}=\beta_{0}+\beta_{1}{\mathrm{AM}}_{ij}+u_{0j}+\varepsilon_{ij}$$

where $PCM_{ij}$ and $AM_{ij}$ are the PCM and AM of interest for fraction i of patient j, respectively. Model fit was summarized using marginal $R_{m}^{2}$ (the anatomical variation in this model) and $R_{c}^{2}$ [25].

## 3. Results

### 3.1. Assessment of Plan Complexity Across the Adaptive Process

The descriptive analysis of PCMs across the database revealed a consistently high level of modulation for all anatomical targets and prescriptions, with an average MU/cGy ≥ 6.8 ± 0.9 and an average ALG ≤ 17.7 mm ± 1.9 mm (see Supplementary Table S1 for more details). Plans generated through the AI-assisted IOE-driven approach showed no statistically significant differences in any of the considered PCMs, neither between the reference and online plans nor across anatomical targets (Table 2). Variance decomposition showed a variable relative contribution from within- and between-patient variance across the evaluated metrics (ICC = 43–79%). While some PCMs exhibited a slight tendency toward within-patient variability (ICC < 0.5, with MU/cGy at 0.43), the majority leaned toward between-patient differences (ICC > 0.5). Overall, this suggests that each PCM captures a slightly different mixture of patient-specific and fraction-specific fluctuations. Notably, LSV stood out as the only metric strongly dominated by between-patient factors (ICC = 0.79). Variance decomposition of PCMs is provided in Figure S1 of the Supplementary Materials.

### 3.2. PSQA Assessment and SPC Framework

**Figure 1:**
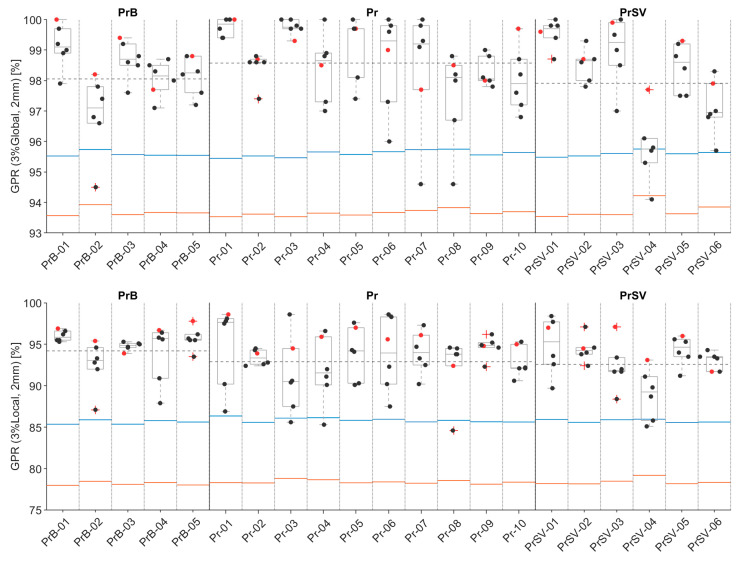
Boxplot of global and local GPR at 3%/2 mm for the three anatomical targets. Blue and red lines represent LOPO-CV-derived TL and AL. Red and black markers represent ‘Reference’ and ‘Online’ plans, respectively.

Using the TG-218 approach, cohort-derived TLs and ALs for the primary clinical endpoint GPR (3%Global, 2 mm) were 95.6% and 93.7%, respectively. Overall, PSQA results showed a mean/median GPR (3%Global, 2 mm) of 98.4%/98.6% (SD = 1.3%, range = [94.1%, 100%], IQR = [97.6%, 99.4%]). LOPO-CV analysis demonstrated overall process stability: for most patients, no fraction fell below the AL (Figure 1), and the LOPO-based TL and AL values were sufficiently stable across iterations to consistently classify the majority of patients as in-control, supporting the feasibility of prospective, cohort-informed SPC monitoring even with a limited institutional dataset.

For GPR (3%Local, 2 mm), cohort-derived TL and AL were 85.8% and 78.4%, respectively, with a mean/median GPR of 93.5%/94.0% (SD = 3.1%, range = [84.6%, 98.6%], IQR = [92.0%, 95.6%]). The LOPO-CV analysis confirmed a comparable pattern of process stability.

### 3.3. Relationship Between Plan Complexity and Delivery Accuracy

The LMEM analysis (Equation (2)) revealed significant associations between several PCMs and GPR (3%Global, 2 mm) after BH-FDR correction: MU/cGy, AAV, MCS, ALG and PI were all significantly associated with the global GPR (*p* ≤ 0.027). Despite statistical significance, the predictive value of individual PCMs remained limited: $R_{m}^{2}$ ranged from 0.006 to 0.122, indicating that plan complexity alone explained at most 12.2% of the variance in GPR. The substantially higher $R_{c}^{2}$ values (0.410–0.501) indicate that an additional 30–49% of variance is captured by the patient-specific random intercept, reflecting baseline patient-level factors not encoded in the PCMs.

**Figure 2:**
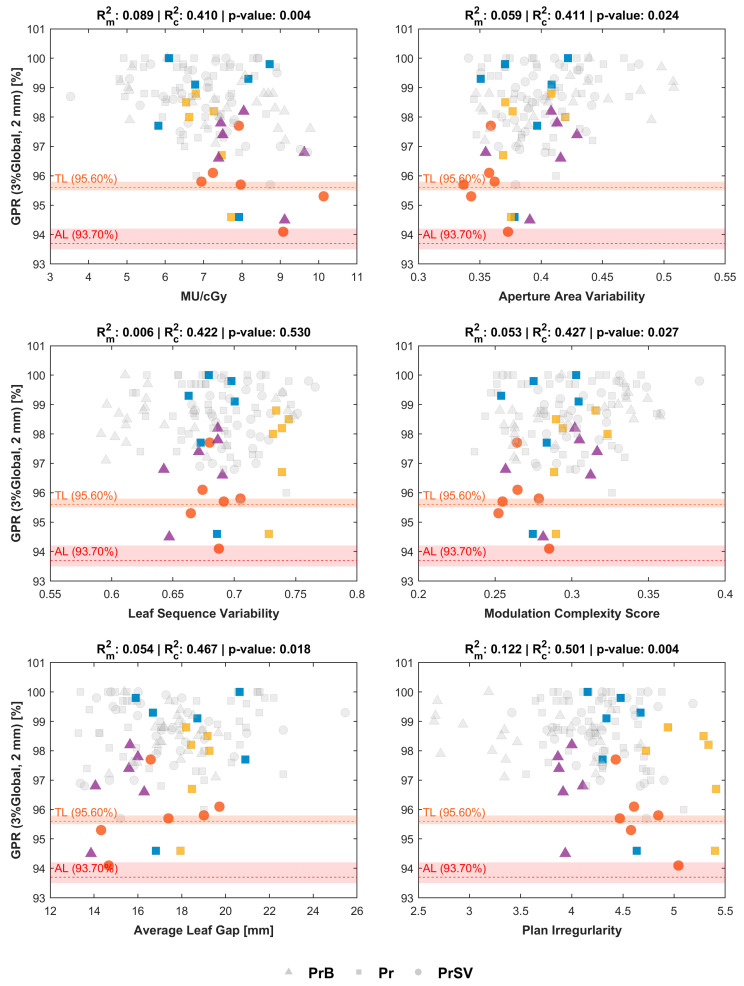
Plan complexity metrics (PCMs) versus GPR (3%Global, 2 mm). Scatter points corresponding to plans. Markers indicate the anatomical target (triangle: prostatic bed; square: prostate w/o seminal vesicles; circle: prostate w/seminal vesicles). Patients with at least one fraction below the TL are indicated using different colors. Horizontal lines denote cohort-derived TL and AL with uncertainty bands derived with LOPO-CV (Leave-One-Patient-Out Cross-Validation) sensitivity analysis. The title of each plot shows the values of $R_{m}^{2}$ and $R_{c}^{2}$ and the corresponding *p*-value of the LMEMs in Equation (2). Statistical significance was assessed using FDR-corrected *p*-values.

The most clinically relevant feature of this relationship is its heteroscedasticity, illustrated in Figure 2. While GPR values are consistently high for low-complexity plans, their dispersion increases as complexity increases, indicating that higher complexity does not uniformly degrade delivery accuracy but renders it increasingly unpredictable. This trend is particularly visible for MU/cGy, ALG and PI. Notably, all fractions failing to meet the TL fell in the high-complexity region for these metrics (MU/cGy > 7, ALG < 20 mm, PI > 4).

The analysis was repeated for GPR (3%Local, 2 mm) and yielded qualitatively consistent findings: MU/cGy, AAV, ALG and PI were significantly associated with local GPR (*p* ≤ 0.024), with comparable heteroscedastic patterns. $R_{m}^{2}$ values were lower (0.003–0.095), as were $R_{c}^{2}$ values (0.104–0.264), consistent with the greater intrinsic variability of local gamma analysis. Results are reported in Figure S2, found in the Supplementary Materials.

### 3.4. Assessment of Anatomical Variations Across the Adaptive Process

According to the results shown in Table 3, no statistically significant anatomical differences exist between the reference and online phases. As expected, significant differences were observed across the three anatomical targets in terms of Bladder ∩ PTV [cm^3^] and Total Overlap [%], reflecting the distinct target–OAR spatial relationships inherent to each group and the associated volume delineation. The high ICC values for PTV Volume and CTV Volume (both 93%) confirm that anatomical variance is mostly attributable to intrinsic patient characteristics, with individual target dimensions remaining stable throughout the treatment. This observation, coupled with the lack of significant differences between the reference and online phases, indicates that the AI-assisted IOE-driven workflow is self-consistent. The lower ICC values for OARs-PTV overlaps highlight the expected intra-patient fluctuations in organ filling, which validates the clinical need for daily adaptive adjustments. The distribution of AMs across patients and details on variance decomposition are illustrated in Supplementary Figure S3. All the descriptive statistics for the anatomical metrics are reported in Table S2, found in the Supplementary Materials.

### 3.5. Relation Between PCMs and AMs

**Figure 3:**
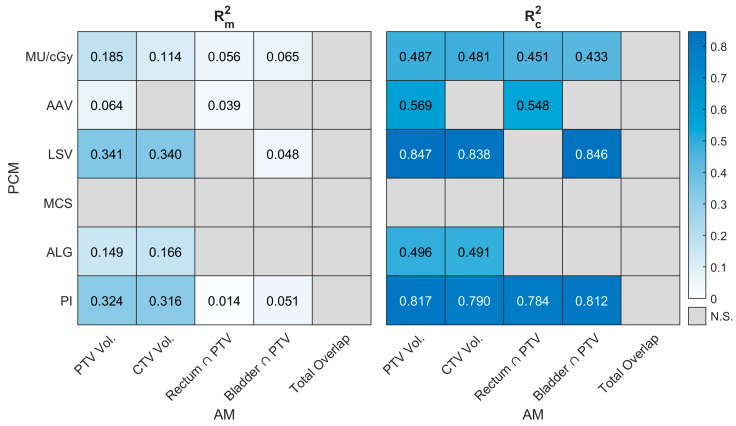
Heatmaps showing the values of $R_{m}^{2}$ and $R_{c}^{2}$ for the LMEM in Equation (4). N.S. = Not significant.

Given the qualitative concordance between global and local GPR results observed in Section 3.3, the PCM–AM association analysis was performed using GPR (3%Global, 2 mm) only. Figure 3 presents $R_{m}^{2}$ and $R_{c}^{2}$ resulting from the LMEM analysis (Equation (4)), quantifying the relation between AMs and PCMs. Overall, $R_{m}^{2}$ values remained below 0.35 (where significant), indicating that anatomical fixed effects explain less than 35% of the total variance in PCMs. This demonstrates that anatomical configurations (such as PTV volume or target–OAR overlaps) do not provide a reliable basis for the prospective estimation of IOE-generated plans modulation. In particular, the influence of the target–OAR overlaps (Rectum ∩ PTV [cm^3^], Bladder ∩ PTV [cm^3^] and Total Overlap [%]) was negligible ($R_{m}^{2}$ 0.065), with none of the evaluated PCMs showing a statistically significant linear relationship with Total Overlap [%].

The high disparity between $R_{m}^{2}$ and $R_{c}^{2}$ values imply that the model’s predictive power is derived almost entirely from the patient-specific random intercept ($u_{0j}$), which represents the baseline complexity established by the optimizer under a fixed set of constraints.

## 4. Discussion

This study investigated the behavior of plan complexity metrics in a CBCT-based online adaptive radiotherapy workflow for prostate cancer treated on the Varian Ethos 1.0 platform. By analyzing plans from three anatomical groups (PrB, Pr, and PrSV), we characterized the patterns of plan complexity in relation to PSQA results and anatomy, and evaluated their implications for a process-based quality assurance.

The IOE consistently produced highly modulated IMRT plans, with elevated MU/cGy and narrow effective apertures (ALG ⪅ 20 mm). No systematic PCM shift was observed between reference and online phases, nor across fractions within a given patient, indicating that once a complexity baseline is established under a fixed set of optimization objectives (directives), it is generally maintained throughout the treatment course. PSQA results were overall satisfactory, with a median global GPR of 98.6% consistently above the cohort-derived tolerance limit of 95.6%. These results are qualitatively consistent with findings by Zhao et al. and Xu et al., who reported near-universal PSQA success for Ethos and Elekta Unity adaptive plans [15,16] and support their suggestion that per-fraction measurement-based PSQA may not be required for every adaptive session [26,27]. However, our results indicate that highly modulated plans exhibit increased delivery instability that is not apparent from aggregate GPR statistics alone. The LOPO-CV analysis demonstrated stable control limits across CV iterations, suggesting that centers initiating oART programs can establish meaningful process thresholds with an initial dataset of modest size. This SPC framework could be interpreted as defining an expected process envelope, complementary to independent dose calculation systems such as Mobius3D.

Although more than one PCMs showed a significant linear relationship with GPR values (either global or local), the low $R_{m}^{2}$ values (≤0.122) confirm that complexity alone is a weak predictor of individual PSQA outcomes, which is consistent with the moderate correlations reported in the literature [9,10]. On the other hand, a clinically relevant finding is the heteroscedasticity of the relationship: GPRs are consistently high for low-complexity plans, but outcome dispersion increases substantially at higher complexity values. In the oART setting, where per-fraction phantom measurement is not feasible, this heteroscedasticity means that PCMs cannot guarantee accuracy, but they can identify fractions operating in a regime where dosimetric outcomes are no longer reliably bounded. This observation, coupled with an estimate of within-patient variance obtained using the LMEM framework, could serve as the basis for the definition of control limits on PCMs, as suggested by Russo et al. [14], in the context of TG-218 SPC. A comparable heteroscedastic pattern was independently reported by Cavinato et al. [12] for MRIdian-based pancreatic SBRT: low-complexity plans achieved uniformly high QA pass rates, while higher-complexity plans showed markedly greater outcome variability. The replication of this finding across two distinct oART platforms and tumor sites strengthens the evidence that PCM-based surveillance can identify fractions operating in a regime of reduced delivery predictability, regardless of the specific optimizer or hardware.

To investigate whether this plan complexity is driven by anatomical variations, we first characterized the inter-fraction anatomical behavior. PTV and CTV remained remarkably stable across fractions (ICC: 93%), while OAR-PTV intersections displayed the expected intra-patient fluctuations driven by daily physiological filling [19,28,29,30]. However, these anatomical variations do not dictate plan complexity: $R_{m}^{2}$ remained below 0.35 across all PCM–AM pairs, and the influence of target–OAR overlap was essentially negligible ($R_{m}^{2}$: 0.000–0.065). The model’s predictive power derives almost entirely from the patient-specific random intercept (representing the baseline complexity established by the IOE under a fixed directive set), around which daily anatomical fluctuations produce only minor oscillations. Practically, since anatomical variations do not explain the observed fluctuations in plan modulation, the IOE should be understood as an optimizer that, within its directive hierarchy, behaves largely autonomously with respect to daily anatomical input, and AMs considered in this study cannot serve as prospective predictors of PCM or PSQA outcomes. Thereby, the high modulation observed could be intended as an intrinsic property of the optimizer’s unconstrained behavior rather than a clinical necessity. Lacking a complexity-limiting constraint, the IOE 1.0 demonstrates a tendency to produce highly modulated plans regardless of the actual geometric challenge posed by the daily anatomy. Whether the complexity control feature introduced in Ethos 2.0 reduces the performance dispersion observed here remains an open question warranting dedicated investigation. This variance structure parallels the findings of Cavinato et al. [12] on the MRIdian platform, where metrics such as TG, ALG, and LeafNum were consistently dominated by between-patient variance (σ^2^_between_ > 74%) across three successive optimizers. The convergent observation across two independent oART platforms (differing in MLC architecture, imaging modality, optimization algorithm, and tumor site) suggests that the predominance of inter-patient variance is a general property of oART-generated plan complexity rather than a platform-specific artifact.

One patient-level deviation was identified. Patient PrSV-04 was independently flagged by both the PSQA LOPO-CV (Figure 1) and the complexity-informed SPC analysis (Figure 2), with two fractions falling below the tolerance limit in the GPR (3%Global, 2 mm) evaluation. Importantly, no daily anatomical anomalies (such as volumetric shifts or unusual OAR overlap) were observed in this patient, suggesting that the elevated modulation stemmed from a challenging baseline geometry rather than fraction-to-fraction instability. This case illustrates how PCMs can serve as a complementary surveillance tool: rather than predicting individual delivery failures, they help identify patients and fractions operating closer to the deliverability limits of a highly automated workflow.

This study is based on a single institution employing nine-field sliding-window IMRT on Ethos 1.0; results may not generalize to VMAT configurations, other oART platforms, or future software versions [31]. Prostate cancer was selected because its structurally stable target geometry allows the IOE’s behavior to be characterized independently of the radical anatomical changes seen at other oART sites (e.g., upper abdomen and thorax), though translation to these districts warrants dedicated investigation. The modest sample size (21 patients, with the smallest subgroup being 5 patients) limits the precision of ICC estimates and subgroup-level interpretations, which remain exploratory. The PSQA subset was enriched for high-complexity fractions by design, precluding direct estimation of per-fraction failure rates. Gamma passing rates were used as the sole delivery accuracy endpoint; while validated for dual-layer MLCs [32], gamma analysis has recognized limitations in detecting clinically meaningful dose differences, and DVH-based endpoints were not evaluated. Finally, intra-fraction motion, delivery time, and secondary dose calculation outputs were not incorporated.

## 5. Conclusions

The IOE of the Varian Ethos platform generates prostate oART plans with consistently high modulation levels that reflect the intrinsic behavior of the AI-assisted optimizer rather than daily anatomical variation. Plan complexity metrics are not reliable predictors of individual delivery outcomes, but the systematic increase in GPR dispersion at higher complexity values supports their use as real-time surveillance indicators within a cohort-informed SPC framework complementary to independent dose calculation for fraction-level verification. Multicenter validation, extension to VMAT and other treatment sites, and integration with DVH-based endpoints are needed to consolidate this approach into a comprehensive QA strategy for oART.

## Figures and Tables

**Table 1:** Patient cohort and fractionation schemes.

Anatomical Target	Number of Patients	Prescription (Total Dose/fx)	Plans Analyzed (Ref/Adapted/Scheduled)	Total Plans (n)
Prostatic bed	3	66.00–72.60 Gy/33 fx	5/134/5	144
	1	60.00–52.60 Gy/20 fx		
	1	52.60 Gy/20 fx		
Prostate w/o seminal vesicles	9	60.00 Gy/20 fx	10/184/1	195
	1	36.25 Gy/5 fx		
Prostate w/seminal vesicles	5	60.00 Gy/20 fx	6/100/6	112
	1	36.00 Gy/6 fx		
*Overall*	*21*	*Multiple regimens (see above)*	*21/418/12*	*451*

**Table 2:** LMEM results: Adjusted *p*-values for the fixed effects of Planning Phase and Anatomical Target.

PCM	Planning Phase *p*-Value	Anatomical Target *p*-Value	ICC
MU/cGy	0.092	0.294	43%
AAV	0.819	0.849	53%
LSV	0.936	0.152	79%
MCS	0.721	0.850	62%
ALG	0.237	0.673	48%
PI	0.701	0.098	69%

**Table 3:** LMEM results: Adjusted *p*-values for the fixed effects of Planning Phase (reference vs. online) and Anatomical Target. The asterisk (*) marks significant comparisons (*p* < 0.05) after FDR correction.

AM	Planning Phase *p*-Value	Anatomical Target *p*-Value	ICC
PTV Vol. [cm^3^]	0.092	0.996	93%
CTV Vol. [cm^3^]	0.070	0.849	93%
Rectum ∩ PTV [cm^3^]	0.996	0.096	65%
Bladder ∩ PTV [cm^3^]	0.996	0.002 *	77%
Total Overlap [%]	0.681	<0.001 *	59%

## Data Availability

The data presented in this study are available on request from the corresponding author.

## Supplementary Materials

The following supporting information can be downloaded at: https://www.mdpi.com/article/10.3390/cancers18101557/s1, Figure S1: Variance decomposition of plan complexity metrics (PCMs) across anatomical target groups; Figure S2: Linear mixed-effects model (LMEM) analysis: Plan complexity metrics (PCMs) versus GPR (3%Local/2 mm); Figure S3: Variance decomposition of anatomical metrics (AMs) across anatomical target groups; Table S1: Descriptive statistics for the plan complexity metrics; Table S2: Descriptive statistics for the anatomical metrics; Selection procedure for PSQA measurements.

## Abbreviations

The following abbreviations are used in this manuscript:

AAV	Aperture Area Variability
AL	Action Level
ALG	Average Leaf Gap
AMs	Anatomical Metrics
AXB	Acuros XB
BH-FDR	Benjamini–Hochberg False Discovery Rate
CBCT	Cone Beam CT
CT	Computed Tomography
CTV	Clinical Target Volume
DVH	Dose-Volume Histograms
FFF	Flattening-Filter-Free
IBSI	Imaging Biomarker Standardization Initiative
ICC	Intraclass Correlation Coefficient
IGRT	Image-Guided Radiation Therapy
IMRT	Intensity-Modulated Radiation Therapy
GPR	Gamma-Passing Rate
IOE	Intelligent Optimization Engine
LMEM	Linear Mixed-Effects Models
LOPO-CV	Leave-One-Patient-Out Cross-Validation
LSV	Leaf Sequence Variability
MCS	Modulation Complexity Score
MLC	Multi-Leaf Collimator
MU	Monitor Unit
oART	Online Adaptive Radiation Therapy
Pr	Prostate Without Seminal Vesicles
PrB	Prostatic Bed
PI	Plan Irregularity
PCMs	Plan Complexity Metrics
PSQA	Patient-Specific Quality Assurance
PrSV	Prostate With Seminal Vesicles
PTV	Planning Target Volume
SPC	Statistical Process Control
TL	Tolerance Level
UCoMX	Universal Complexity Metrics Extractor
VMAT	Volumetric Modulated Arc Therapy
