# Supplementary material for: Characterization of Plan Complexity and Its Role in Quality Assurance for AI-Assisted CBCT-Based Online Adaptive Radiotherapy of Prostate Cancer
Source: Cancers (Basel). 2026 May 11;18(10):1557. doi: 10.3390/cancers18101557 (PMC13204234; doi:10.3390/cancers18101557)
Supplement: Supplementary file 1 [file cancers-18-01557-s001.zip › cancers-4202234-supplementary.pdf]

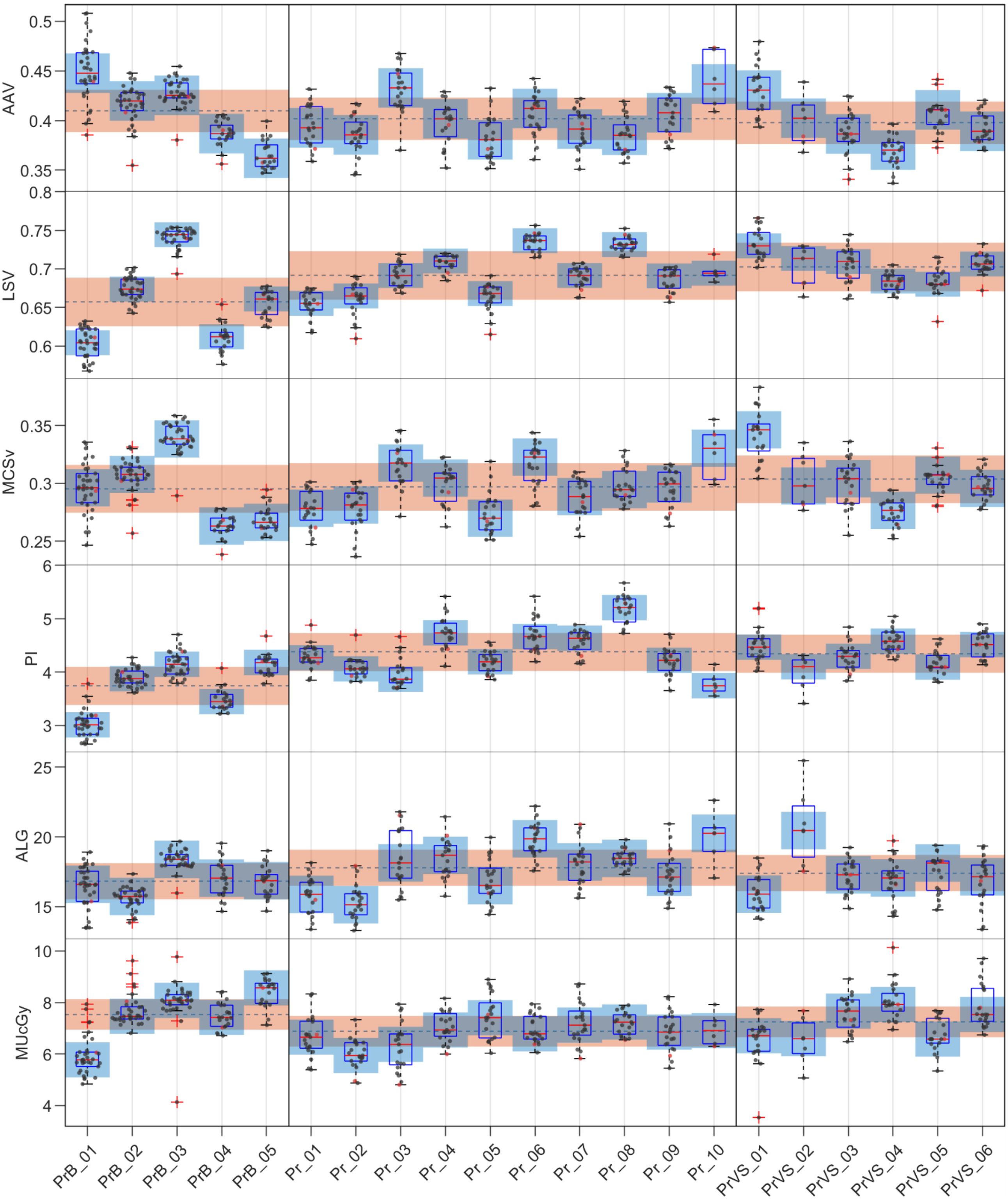

Figure S1. Variance decomposition of plan complexity metrics (PCMs) across anatomical target groups. Boxplots of PCMs categorized by anatomical target group: Prostatic Bed (PrB) , Prostate without seminal vesicles (Pr), and Prostate with seminal vesicles (PrSV). The blue and red bands represent the within-patient (σ_w) and between-patient (σ_w) variability, respectively, derived from linear mixed-effects models (LMEMs) fitted independently for each group.

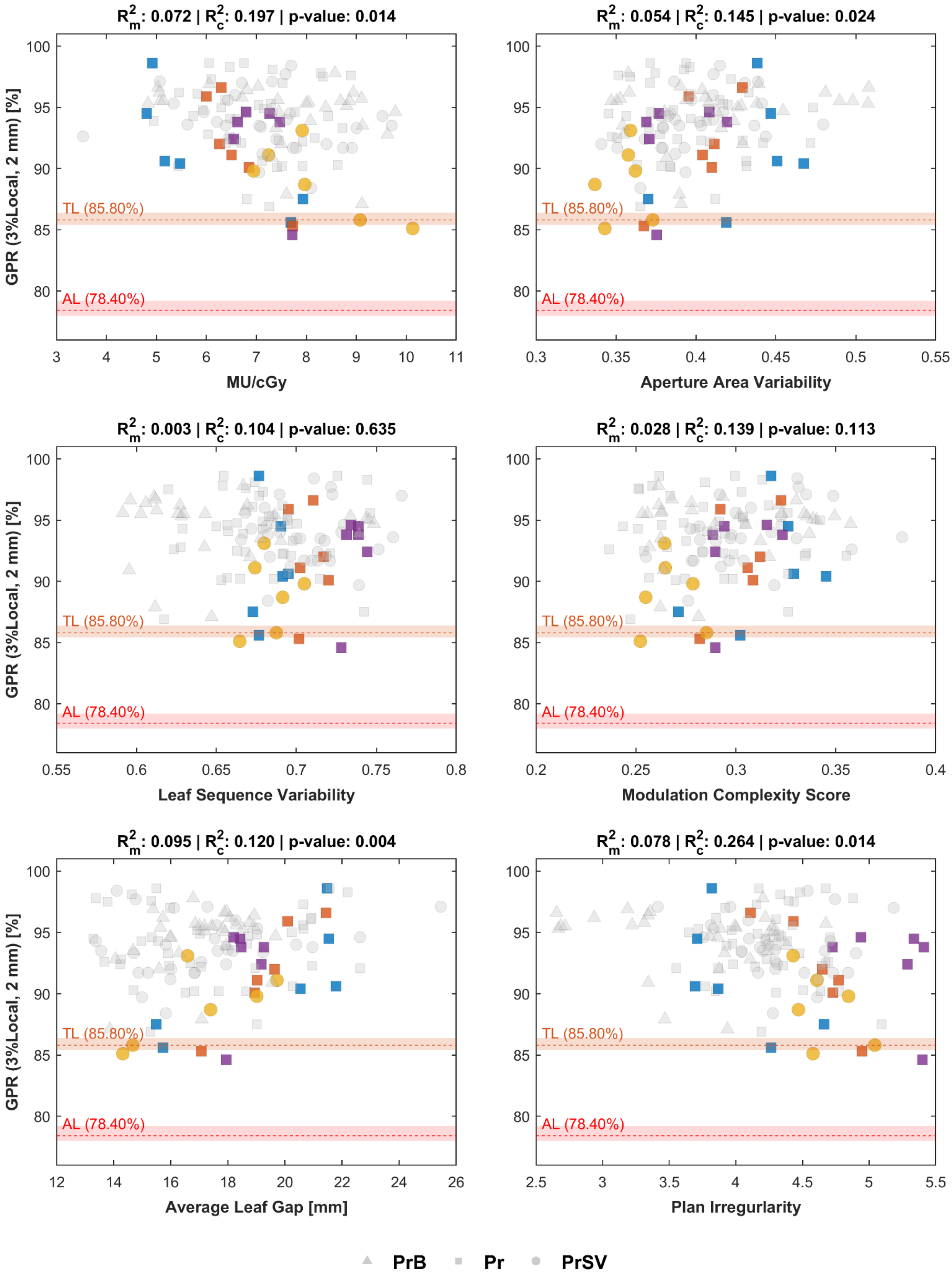

Figure S2. Plan complexity metrics (PCMs) versus GPR (3%Local, 2 mm). Scatter points corresponding to plans. Markers indicate the anatomical target (triangle: prostatic bed; square: prostate w/o seminal vesicles; circle: prostate w/ seminal vesicles). Patients with at least one fraction below the TL are indicated using different colors. Horizontal lines denote cohort-derived TL and AL with uncertainty bands derived with LOPO-CV (Leave-One-Patient-Out Cross-Validation) sensitivity

analysis. The title of each plot shows the values of R^2m and R^2c and the corresponding p-value of the LMEMs in Eq. (2). Statistical significance was assessed using FDR-corrected p-values.

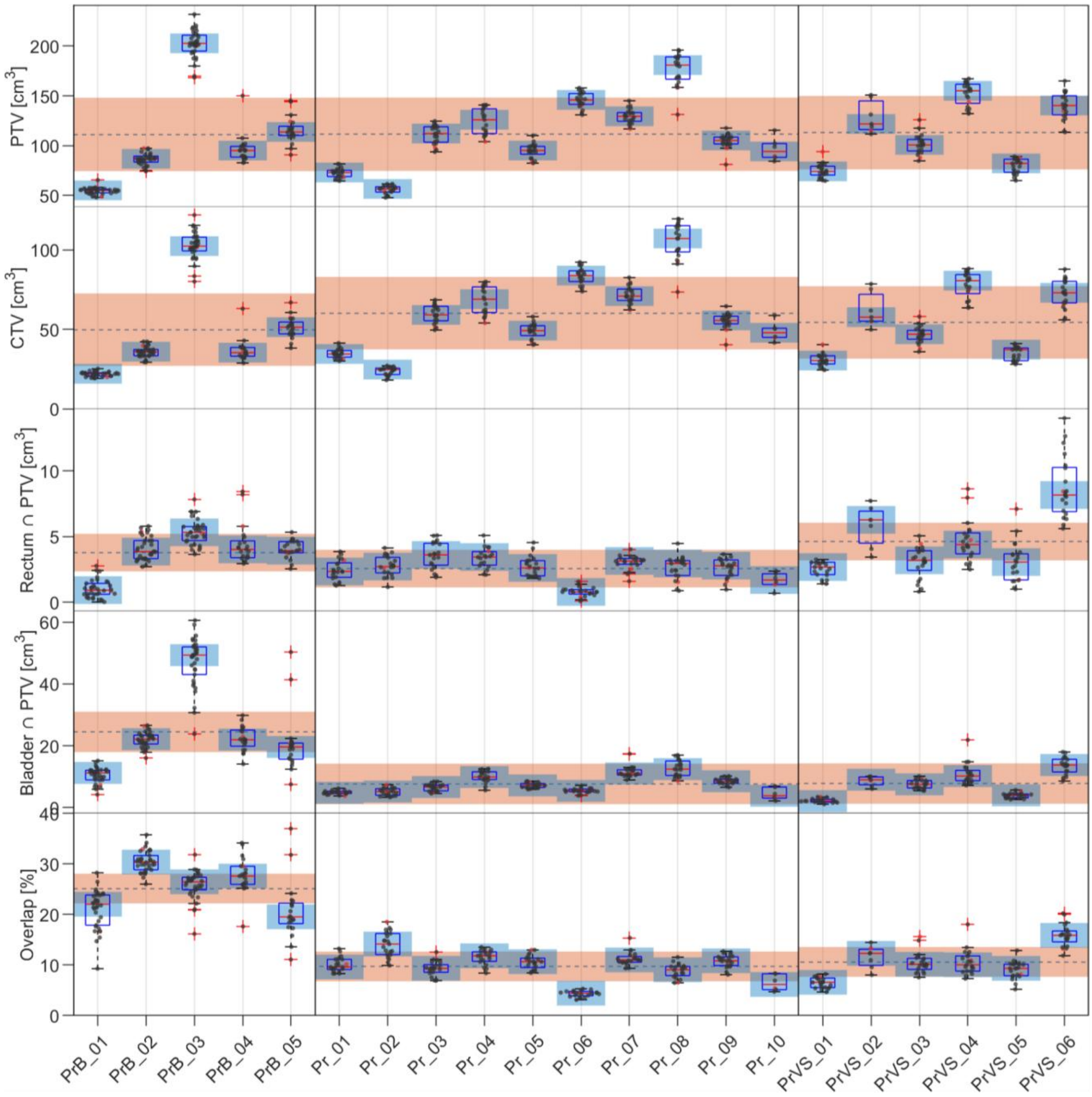

Figure S3. Variance decomposition of plan anatomical metrics (AMs) across anatomical target groups. Boxplots of AMs categorized by anatomical target group: Prostatic Bed (PrB), Prostate without seminal vesicles (Pr), and Prostate with seminal vesicles (PrSV). The blue and red bands represent the within-patient (σ_w) and between-patient (σ_w) variability, respectively, derived from linear mixed-effects models (LMEMs) fitted independently for each group.

Table S1. Descriptive statistics for the plan complexity metrics.

PCM	Mean	SD	Median	Range [min-max]	IQR [1st-3rd qrtl]
PrB					
MU/cGy	7,4	1,0	7,4	4,8 - 9,8	6,8 - 8,1
AAV	0,42	0,03	0,40	0,35 - 0,51	0,39 - 0,44
LSV	0,66	0,05	0,67	0,57 - 0,75	0,61 - 0,69
MCS	0,30	0,03	0,30	0,24 - 0,36	0,28 - 0,32

ALG [mm]	16,8	1,3	16,4	13,5 - 19,7	15,8- 17,9
PI	3,8	0,4	3,9	2,7 - 4,7	3,5 - 4,1
Pr					
MU/cGy	6,8	0,9	6,8	4,8 - 8,9	6,2 - 7,5
AAV	0,40	0,03	0,40	0,35 - 0,45	0,38 - 0,42
LSV	0,69	0,03	0,68	0,61 - 0,76	0,66 - 0,72
MCS	0,30	0,03	0,29	0,24 - 0,36	0,28 - 0,32
ALG [mm]	17,7	1,9	17,5	13,3 - 22,6	16,0 - 19,0
PI	4,5	0,5	4,4	3,6 - 5,7	4,1 - 4,8
PrSV					
MU/cGy	7,2	1,1	7,3	3,5 - 10,1	6,6 - 7,9
AAV	0,40	0,03	0,40	0,34 - 0,48	0,38 - 0,42
LSV	0,70	0,03	0,70	0,63 - 0,77	0,68 - 0,72
MCS	0,30	0,03	0,30	0,25 - 0,38	0,28 - 0,33
ALG [mm]	16,9	1,9	16,9	13,4 - 25,5	15,8 - 18,0
PI	4,4	0,3	4,4	3,4 - 5,2	4,2 - 4,6

Table S2. Descriptive statistics for the anatomical metrics.

AM	Mean	SD	Median	Range [min-max]	IQR [1st-3rd qrtl]
PrB					
PTV Vol. [cm^3]	111,5	51,2	90,7	48,1 - 231,9	81,3 - 169,9
CTV Vol. [cm^3]	50,07	26,1	37,4	19,2 - 121,9	33,2 - 80,1
Rectum ∩ PTV [cm^3]	3,85	1,6	3,7	0,01 - 8,4	2,8 - 5,1
Bladder ∩ PTV [cm^3]	23,5	14,3	20,6	4,2 - 60,5	11,6 - 30,7
Total Overlap [%]	25,6	4,8	26.0	9,3 - 37.0	22,3 - 28,8
Pr					
PTV Vol. [cm^3]	111,2	32,5	111,1	47,8 - 196.0	84,4 - 139,1
CTV Vol. [cm^3]	58,1	21,2	57,4	18,2 - 119,6	43,2 - 73,3
Rectum ∩ PTV [cm^3]	2,4	1,1	2,4	0,1 - 5,1	1,5 - 3,3
Bladder ∩ PTV [cm^3]	7,6	3,1	6,8	3,1 - 17,4	5,2 - 9,9
Total Overlap [%]	9,6	2,8	9,4	3,1 - 18,5	8,3 - 11,4
PrSV					
PTV Vol. [cm^3]	110,6	31,4	102,2	64,7 - 167,2	82,7 - 140,3
CTV Vol. [cm^3]	54,2	18,5	48,4	24,5 - 88,2	37,8 - 72,1
Rectum ∩ PTV [cm^3]	5,3	2,8	4,1	0,80 - 14,0	3,1 - 7,2
Bladder ∩ PTV [cm^3]	9,1	4,2	8,1	1,1 - 21,9	6,2 - 11,8
Total Overlap [%]	11,5	3,5	10,6	4,7 - 20,2	8,3 - 14,5

Selection procedure for PSQA measurements.

The selection procedure was designed to systematically sample both anomalous and normal fractions based on plan complexity. This was done separately for each anatomical group. An overall anomaly score was created to combine the information provided by the different complexity metrics (L2-norm of the scores obtained by standardizing the metric values using the median and interquartile range). Fractions were then classified as 'Extreme' if any metric fell outside the 2.5th–97.5th percentile range, and 'Bulk' otherwise. For each patient, five adapted fractions were selected, prioritizing ‘Extreme’ sessions ranked by their anomaly score, followed by a random selection of ‘Bulk’ sessions. This stratified approach ensured that the dataset was sufficiently enriched with the highest-complexity plans, while still maintaining a baseline of standard fractions for comparison.
